# Cytogenetic and genetic data support *Crossodactylus aeneus* Müller, 1924 as a new junior synonym of *C. gaudichaudii* Duméril and Bibron, 1841 (Amphibia, Anura)

**DOI:** 10.1590/1678-4685-GMB-2020-0301

**Published:** 2021-03-22

**Authors:** Stenio Eder Vittorazzi, Luciana Bolsoni Lourenço, Michelle Louise Zattera, Luiz Norberto Weber, Shirlei Maria Recco-Pimentel, Daniel Pacheco Bruschi

**Affiliations:** 1 Universidade do Estado de Mato Grosso, Faculdade de Ciências Biológicas, Agrárias e da Saúde, Departamento de Ciências Biológicas, Tangará da Serra, MT, Brazil.; 2 Universidade Federal do Paraná, Setor de Ciências Biológicas, Departamento de Genética, Programa de Pós-Graduação em Genética, Curitiba, PR, Brazil.; 3 Universidade Estadual de Campinas, Instituto de Biologia, Departamento de Biologia Estrutural e Funcional, Campinas, SP, Brazil.; 4 Universidade Federal do Sul da Bahia, Instituto Sosígenes Costa de Humanidades, Artes e Ciências, Porto Seguro, BA, Brazil.

**Keywords:** Crossodactylus, chromosome, karyotype, synonymous species, species delimitation

## Abstract

The nominal anuran species *Crossodactylus gaudichaudii* Duméril and Bibron, 1841 and *Crossodactylus aeneus* Müller, 1924 are indistinguishable based on adult and larval morphology, being subject of taxonomic doubts. Here, we describe the karyotypes of *C. gaudichaudii* and *C. aeneus*, using classical and molecular cytogenetic markers. In addition, we used sequences of the H1 mitochondrial DNA to infer their phylogenetic relationships by Maximum Likelihood (ML) and Maximum Parsimony (MP) approaches and species delimitation test (by bPTP approach). The karyotypic data do not differentiate *C. gaudichaudii* and *C. aeneus* in any of the chromosome markers assessed. In both phylogenetic analyses, *C. gaudichaudii* and *C. aeneus* were recovered into a strongly supported clade. The species delimitation analysis recovered the specimens assigned to *C. gaudichaudii* and *C. aeneus* as a single taxonomic unit. Taken the cytogenetic and genetic results together with previous studies of internal and external morphology of tadpoles and biacoustic pattern, *C. gaudichaudii* and *C. aeneus* could not be differentiated, which supports the hypothesis that they correspond to the same taxonomic unit, with *C. aeneus* being a junior synonym of *C. gaudichaudii.*

## Introduction

The genus *Crossodactylus* (Hylodidae) includes 14 species of diurnal frogs that inhabit streams banks, ranging from Alagoas state in northeastern Brazil to Rio Grande do Sul state in southern Brazil, and being found in southern Paraguay and northern Argentina (Carcerelli and Caramaschi, 1993; Frost, 2020). Historically, the taxonomic investigation of the *Crossodactylus* species has been based on phenotypic features, that is, the external and internal morphology of adults and larvae, bioacoustics, and morphometric parameters (Caramaschi and Sazima, 1985; Pimenta et al., 2014; 2015). Although species of *Crossodactylus* were included in some molecular phylogenetic inferences (e.g., Pyron and Wiens, 2011; Grant et al., 2017), a phylogenetic analysis focused on this genus remain to be done.

Based on the morphological and morphometric evidence, Caramaschi and Sazima (1985) recognized three *Crossodactylus* species groups, the *Crossodactylus gaudichaudii*, *Crossodactylus trachystomus*, and *Crossodactylus schmidti* (monotypic) species groups. However, Pimenta et al. (2014) questioned the validity of the analysis of morphometric characters in this genus, given that many characters overlap extensively between species (except in *Crossodactylus grandis*). Because of the phenotypic similarities of the *Crossodactylus* species, more reliable and conclusive taxonomic studies will require the systematic integration of morphological and molecular evidence.

One clear example of this taxonomic dilemma is found in the two species of the *C. gaudichaudii* group, *C. gaudichaudii* and *C. aeneus*, which have overlapping geographic ranges in southeastern Brazil, where they occur predominantly in the states of São Paulo and Rio de Janeiro (Frost, 2020). The original description of *C. gaudichaudii* lacks details on the type locality, which was identified only as “Brazil” (Duméril and Bibron, 1841; Guibé, 1948), although Bokermann (1966) suggested that the city of Rio de Janeiro was the most probable type locality of the species. The type locality of *C. aeneus* is given as “Barreira” in the Serra dos Órgãos range (Müller, 1924), locality currently belonging to the municipality of Guapimirim, in Rio de Janeiro state, Brazil. The uncertainties with regard to the geographic distribution of these species have been magnified by overlapping bioacoustic parameters (Pimenta et al., 2008, 2015) and both the external (Francioni and Carcerelli, 1993; Silva-Soares et al., 2015) and internal oral morphology of the tadpoles (Weber and Caramaschi, 2006; Silva-Soares *et al*., 2015). These characters have failed to provide reliable diagnostic traits that confirm their taxonomic status as independent evolutionary lineages (Faivovich, 1998; Weber and Caramaschi, 2006; Silva-Soares *et al*., 2015).

To answer if *C. aeneus* is a valid species, we aimed to contribute to the assessment of this taxonomic problem comparing *C. gaudichaudii* and *C. aeneus* based on a detailed characterization of their karyotypes and on genetic analyses of H1 mitochondrial DNA sequences (12S+tRNA-val+16S).

## Material and Methods

### *Crossodactylus aeneus* and *C. gaudichaudii* sampling 

We sampled the type locality of *C. aeneus* and the city of Rio de Janeiro, which is the most probable type locality of *C. gaudichaudii* (see Bokermann, 1966). Three adult *C. gaudichaudii* specimens (ZUEC 17569-17571) were collected from Parque Lage in the Tijuca Forest in the municipality of Rio de Janeiro, Rio de Janeiro state, Brazil (22°57’29” S, 43°12’38” W, 129 m), and one topotype of *C. aeneus* (tadpole, ZUEC 20459) was collected from Barreira, near the Soberbo River in the municipality of Guapimirim, Rio de Janeiro state, Brazil (22°29’19” S, 43°00’43” W, 582 m).

**Figure 1 -:**
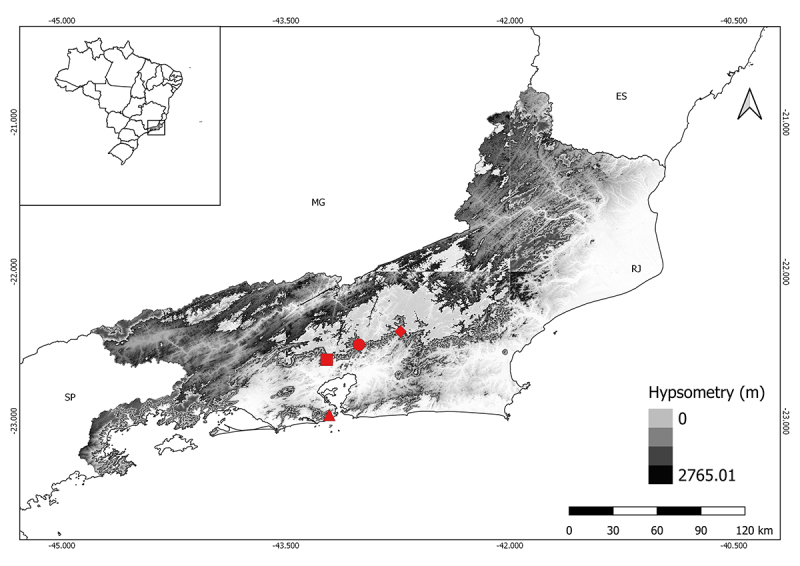
Localities of the specimens assigned as *C. gaudichaudii* and *C. aeneus* analyzed in the present work. ▲ *Crossodactylus gaudichaudii* from Parque Lage, Rio de Janeiro city (probable type locality of this species - see text for detail); ■ *Crossodactylus aeneus* from the Taquara Municipal Natural Park (PNMT), municipality of Duque de Caxias; ● *Crossodactylus aeneus* (topotype) from Barreira, municipality of Guapimirim; and ◆ *Crossodactylus aeneus* from Reserva Ecológica de Guapiaçu, Cachoeiras de Macacu (Amaral et al., 2019).

We also analyzed three adult specimens (ZUEC 17578-17580) from the Parque Natural Municipal da Taquara (PNMT) in the municipality of Duque de Caxias, Rio de Janeiro state, Brazil (22°35’23” S, 43°13’38” W, 241 m) (Figure 1). These specimens were compared with specimens from collections (Table S1), original descriptions of *C. gaudichaudii* and *C. aeneus*, and literature information about the species occurrence. PNMT are located on the geographical region named “Serra dos Órgãos”, slope of Petrópolis, the same mountain region of the type locality of *C. aeneus* (Figure 1). Hence, because no morphological distinction was noted between the specimens from Duque de Caxias and those from the type locality of *C. aeneus* or, those specimens used for this species description, we tentatively assigned to *C. aeneus* the specimens from Duque de Caxias.

The collection of specimens was authorized by the Brazilian Institute for the Environment and Renewable Natural Resources (IBAMA - Process number 21619-1). All collected specimens were fixed and deposited in the Museum of Zoology “Professor Adão José Cardoso” of the University of Campinas (ZUEC), in Campinas, São Paulo, Brazil. Details of the location and voucher number information is summarized in Table S2. For the analyses described below, tissue samples were extracted from specimens anesthetized with 5% Lidocaine (applied to the skin), following the recommendations of the Herpetological Animal Care and Use Committee (HACC) of the American Society of Ichthyologists and Herpetologists (available at http//www.asih.org).

### Extraction of the DNA

The genomic DNA was extracted from liver or muscle tissue, previously maintained at -80 °C, from three *C. gaudichaudii* specimens and four *C. aeneus* (Table S2). The tissue was lysed in TNES (50 mM Tris-HCl, pH 7.5, 400 mM NaCl, 20 mM EDTA, and 0.5% SDS) supplemented with proteinase K (100 μg/mL) at 56 °C for approximately 3 hours. After lysis, the samples were treated with RNAse (50 μg/mL), and NaCl was added to a final concentration of ~1.7 M. The DNA was precipitated in isopropyl alcohol, washed in ethanol (70%), and rehydrated in TE (10 mM Tris-HCl, 1 mM EDTA, pH 8). For quality control and to quantify the genomic DNA, the samples were electrophoresed in 0.8% agarose gel and analyzed by spectrophotometry.

### Mitochondrial DNA sequencing

To generate data for the genetic distance, phylogenetic, and species delimitation analyses, sequences of the H1 mitochondrial DNA (that comprise the 12S rRNA, Val-tRNA, and 16S rRNA genes) were obtained by PCR using the primer pairs MVZ 59 (Graybeal, 1997)/Titus I (Titus and Larson, 1996) and 12L13 (Feller and Hedges, 1998)/16Sbr (Palumbi et al., 2002). The amplified products were electrophoresed in 1% agarose gels and then purified using the GFX PCR and Gel Band DNA Purification kit (GE Healthcare) according to the manufacturer’s instructions. The samples were sequenced using the BigDye Terminator kit (Applied Biosystems), with the primers MVZ 59, MVZ 50 (Graybeal, 1997), 12L13 (Feller and Hedges, 1998), Titus I (Titus and Larson, 1996), 16L2a, 16H10 (Hedges, 1994), 16sAR, and 16sBR (Palumbi *et al*., 2002).

The products of the sequencing reactions were purified by precipitation in 80% ethanol and centrifugation at 1,200 rpm for 30 minutes, and were then washed in 70% ethanol and centrifuged for 10 minutes. Once dried, the products were resuspended in loading dye (Blue-Dextran-EDTA/Formamide, 1:5), denatured for 3 minutes at 94 °C, and then transferred to automatic sequencer. The sequences were edited using the Bioedit software, available at http://www.jwbrown.mbio.ncsu.edu/BioEdit/bioedit.html (Hall, 1999).

### DNA sequence analyses

The mitochondrial DNA dataset included sequences from three individuals of *C. gaudichaudii* and four *C. aeneus,* and all the H1 or partial 16S gene sequences available in the GenBank for *Crossodactylus* species (Table S2), including three partial sequences of the 16S rRNA gene of *C. aeneus* from Reserva Ecológica de Guapiaçu, Cachoeiras de Macacu, Rio de Janeiro, Brazil (Amaral et al., 2019; Figure 1). Therefore, we included sequences of *C. aeneus, C. caramaschii* (*C. gaudichaudii* species group), *C. werneri* (*C. dispar* species complex), *C. trachystomus* (*C. trachystomus* species group), and *C. schmidti* (*C. schmidti* species group). We also included *Megaelosia goeldii* and *Hylodes phylodes* as representatives of the two other genera comprised in the Hylodidae family, and representatives of Alsodidae, which has been inferred as the sister group of Hylodidae (Pyron and Wiens, 2011; Grant et al., 2017) (for details, see Table S2). The dataset was aligned using the MAFFT v7 application (Katoh et al., 2019) and generated a matrix composed of the 2,369 bp.

The phylogenetic analyses were based on the Maximum Likelihood (ML) and Maximum Parsimony (MP) approaches. For the ML analysis, the GTR substitution model was inferred by MrModeltest v2.3 (Nylander, 2004) as the best model of evolution. The unpartitioned DNA matrix sequence was implemented in RAxML (Kozlov et al., 2019) with estimate stationary base frequencies, executing 10 separate searches with different starting trees. The bootstrap analysis was performed using 100 replicates to assess the statistical support of clades. The MP analyses were conducted in TNT v1.5 (Goloboff and Catalano, 2016) using the new technology search option (the best length was hit 100 times), including sectorial searches, ratchet, tree drifting, and tree fusing. The gaps were considered as fifth state and support of the edges was evaluated by bootstrap analysis with 1,000 replicates.

Uncorrected p-distances among and within clades of interest were calculated using MEGA X (Kumar et al., 2018). This analysis was conducted with the mitochondrial H1 and also with the partial fragments of the 16S rRNA gene. Gaps and missing data were deleted in pairwise comparisons.

To assess the taxonomic status of *C. gaudichaudii* and *C. aeneus*, we used the cladogram inferred in the RAxML analysis to employ a tree-based species delimitation test, using the Poisson Tree Process (PTP) model (Zhang et al., 2013). We used the bPTP version of the PTP method, available on the webserver (http://species.h-its.org/ptp/). The bPTP analysis was run with all parameters set at default except the MCMC, which was set at 500,000 generations. The outgroup was removed to improve the delimitation results as suggest by server.

### Classical cytogenetic preparations

Mitotic metaphases were obtained from cell suspensions of the intestinal epithelium from three *C. gaudichaudii* specimens and four *C. aeneus* (Table S2) previously treated with colchicine (King and Rofe, 1976, with modifications from Gatto et al., 2018). The chromosomes were stained with Giemsa (10%) and then C-banded (King, 1980). The slides were processed using the Ag-NOR method (Howell and Black, 1980) to detect the Nucleolus Organizer Regions (NORs). The metaphasic chromosomes were photographed under an Olympus BX-60 microscope and classified according to Green and Sessions (1991).

### Fluorescent *in situ* hybridization (FISH) 

The FISH experiments were carried out on specimens ZUEC 17569 and ZUEC 17579 (Table S2), which represent the populations of Rio de Janeiro and Duque de Caxias, respectively. The PcP190 satellite DNA sequence previously isolated from *C. gaudichaudii* by Vittorazzi et al. (2014) was amplified to obtain chromosomal probes. For this, one cloned fragment was amplified by PCR in the presence of digoxigenin-dUTP (Roche) and primers T7 and SP6, which flank the connection site of the pGEM-T Easy Vector (Promega). The probes were mixed with salmon DNA (1 ng/μL of probe) and precipitated in ethanol. The DNA was dissolved in a hybridization buffer at pH 7 composed of deionized formamide (50%), 2 x SSC, phosphate buffer (40 mM), Denhardt’s solution, SDS (1%), and dextran sulfate (10%). The *in situ* hybridization technique was based on Viegas-Péquignot (1992), with modifications for the detection of digoxigenin-labeled probes with anti-DIG-Rhodamine (Roche).

The microsatellites (CA)_15_ and (GATA)_8_ oligonucleotides were marked directly with Cy5-fluorochrome at the 5’ end during synthesis (Sigma-Aldrich) and used as probes in FISH assays that followed the protocol of Kubat et al. (2008), under high stringency (77%) conditions. Images of the hybridized metaphase chromosomes were captured with an Olympus BX-60 microscope and edited with the Image-Pro Plus program (Media Cybernetics).

## Results

### Phylogenetic inferences and species delimitation based on mitochondrial DNA sequences

**Figure 2 -:**
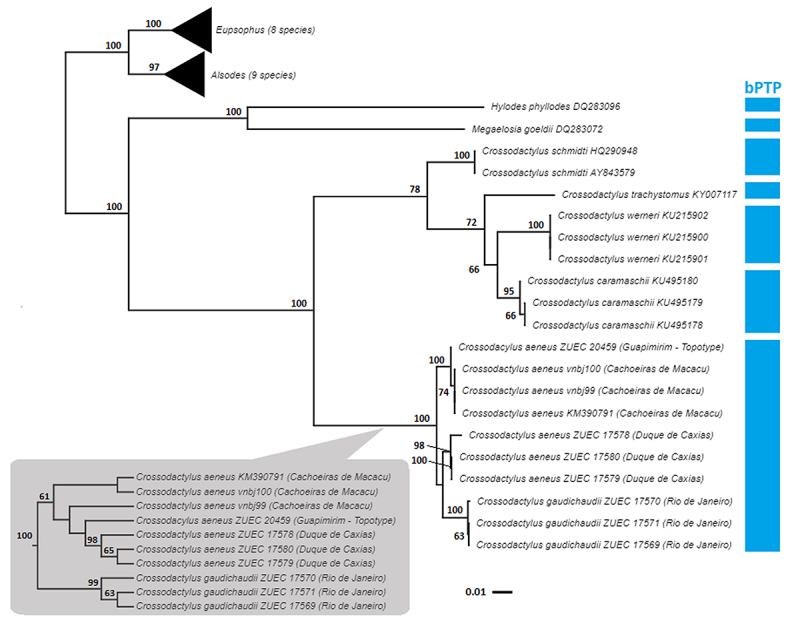
Phylogenetic relationships among the *Crossodactylus* species analyzed in the present study, inferred by maximum likelihood analysis of a 2,369-bp H1 mitochondrial DNA sequence matrix. The gray box shows the clade of *C. gaudichaudii* and *C. aeneus* obtained in the Maximum Parsimony analysis. Note the incongruence recovered to the specimens from Duque de Caxias (for complete tree, see Figure S1). The numbers at each node indicate the bootstrap values (values below 50 have been omitted). The blue blocks indicate the partitions recovered by the bPTP analysis of the *Crossodactylus* specimens.

All *Crossodactylus* species were recovered into one strongly supported sister-clade of *Hylodes phyllodes* + *Megaelosia goeldii* in the ML and MP analyses. The species *C. werneri*, *C. trachystomus*, *C. caramaschii* and *C. schmidti* formed the sister-group of a clade composed of *C. gaudichaudii* and *C. aeneus*, however, with low bootstrap support (Figure 2 and Figure S1).

The clade containing *C. gaudichaudii* and *C. aeneus* was strongly supported in both analyses. The *C. gaudichaudii* specimens (from Rio de Janeiro city) and those specimens of *C. aeneus* from Guapimirim and Cachoeiras de Macacu formed two distinctive groups in both phylogenetic inferences; however, the relationships of *C. aeneus* from Duque de Caxias differed in both analyses. In the ML analysis, the three *C. aeneus* specimens from Duque de Caxias were grouped together with those three *C. gaudichaudii* specimens, however, without bootstrap support (Figure 2). In contrast, in the MP analysis, *C. aeneus* was recovered as monophyletic, with all the specimens assigned to this species clustered together in a low supported clade (61% of bootstrap - Figure 2).

The genetic distances between *C. gaudichaudii* and *C. aeneus* were low, with average uncorrected p-distance of 1.7% to the partial 16S (minimum 1.5% and maximum 1.9%) and 2% to H1 sequence (minimum 1.8% and maximum 2.3%) (average values in Table 1). Genetic distances within *C. aeneus* ranged from 0% to 0.8% in 16S sequence while within the *C. gaudichaudii* population ranged from 0% to 0.1% in 16S sequences. As the specimens of *C. aeneus* from Duque de Caxias grouped differently in the ML and MP analyses, we also considered these specimens separately in additional comparisons. In 16S sequences, the specimens from Duque de Caxias differed from the remaining *C. aeneus* specimens in 0.8%, and from *C. gaudichaudii* in 1.5%. The average genetic distance of *C. gaudichaudii* and *C. aeneus* against the other species included in the analysis (*C. werneri*, *C. trachystomus*, *C. caramaschii* and *C. schmidti*) ranged from 7.4% to 10.7% in 16S sequences (Table 1).

**Table 1 -:** Uncorrected p-distances (in percentage) based on partial 16S rDNA (bottom triangle) and H1 mitochondrial DNA (top triangle) of the *Crossodactylus* species analyzed in the present study. Gray cells show intraspecific variation of the partial 16S rDNA (left) and H1 mitochondrial DNA (right).

Species	1	2	3	4	5	6	7	8
1. *C. aeneus* (Guapimirim - Topotype)	-/-	1.6	-	2	10.4	-	-	-
2. *C. aeneus* (Duque de Caxias)	0.7	0/0.5	-	2	10.4	-	-	-
3. *C. aeneus* (Cachoeiras de Macacu)	0.1	0.8	0.1/-	-	-	-	-	-
4. *C. gaudichaudii* (Rio de Janeiro city)	1.7	1.5	1.8	0/0.1	10.2	-	-	-
5. *C. schmidti*	7.4	7.6	7.6	7.4	0/0	-	-	-
6. *C. trachystomus*	8.6	8.2	8.6	8.2	6.7	-/-	-	-
7. *C. caramaschii*	9.2	8.7	9.2	7.4	6.8	6.4	0.2/-	-
8. *C. werneri*	10.5	10.7	10.4	9.6	6.3	6.5	5.6	0/-

The bPTP species delimitation method recognized *C. caramaschii*, *C. trachystomus*, *C. werneri* and *C. schmidti* as independent taxonomic units, which reinforces the capacity of this procedure to delimit species of *Crossodactylus* (Figure 2). In addition, the bPTP approach recovered *C. gaudichaudii* and *C. aeneus* as a single partition, with a Bayesian support of 0.94, supporting the hypothesis that the specimens assigned to these species belong to a single species (Figure 2).

### Cytogenetic analysis

**Figure 3 -:**
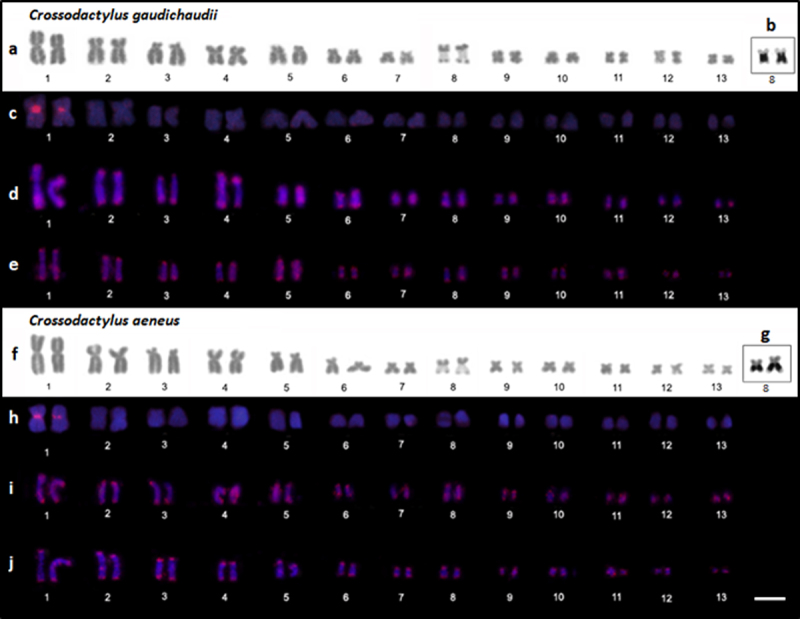
Karyotypes of *C. gaudichaudii* and *C. aeneus.* Chromosomes stained with Giemsa (a, f); The secondary constrictions in the long arm of pair 8 coincide with the NOR, which were silver-impregnated by the Ag-NOR method, as shown in insets b and g. Dark planks show chromosome hybridized with probes for the PcP190 satellite DNA (c, h), (CA)_15_ microsatellite repeat (d,i), and (GATA)_8_ microsatellite repeat in (e,j). Bar = 10 µm.

The specimens assigned to both *C. gaudichaudii* and *C. aeneus* had a diploid number of 2n = 26 chromosomes, with a karyotype composed of six metacentric pairs (1, 4, 9, 11-13), five submetacentric pairs (2, 6-8 and 10), and two subtelocentric pairs (pairs 3 and 5) (Figure 3a, f). An extensive secondary constriction was observed in the long arm of the homologs of pair 8 in some metaphases, which coincides with NOR. On the other hand, smaller secondary constrictions were also seen and consequently, in these cases, the NOR-bearing chromosome pair could be classified as pair 8 according to its size. Therefore, despite a secondary constriction increased the chromosome size in most metaphases, we classified the NOR-bearing chromosome pair as pair 8 to reflect our hypothesis of chromosomal homology when we compare the karyotypes described here with karyotypes previously described to other *Crossodactylus* species (Beçak, 1968; De Lucca et al., 1974; Aguiar-Jr et al., 2004; Amaro, 2005). The C-banding technique detected a weak centromeric heterochromatin signal in some of the chromosome pairs of both karyotypes. As the C-banding data were insufficient for discussion, we show these results in Figure S2.

A conspicuous PcP190 satellite DNA cluster was found in the centromeric region of the homologs of pair 1 in both species (Figure 3c, h), while the mapping of the microsatellite repeats (CA)_15_ (Figure 3d,i) and (GATA)_8_ (Figure 3e,j) revealed hybridization signals in the terminal regions of all the chromosomes in all the specimens assigned to both *C. gaudichaudii* and *C. aeneus*.

## Discussion

Chromosomal analysis of *C. gaudichaudii* and *C. aeneus* showed the same diploid (2n) and fundamental (FN) numbers previously reported to *C. caramaschii* (Aguiar-Jr et al., 2004; Amaro, 2005), *C. dispar* (De Lucca et al., 1974), *C. grandis* (Beçak, 1968) and *C. schmidti* (Amaro, 2005), suggesting an overall similarity among the *Crossodactylus* karyotypes. The NOR located in a small-sized biarmed chromosome pair is a common feature within *Crossodactylus* species, as NORs were detected on the long arm of pair 8 in karyotypes of *C. caramaschii* (Aguiar-Jr *et al*., 2004), *C. schmidti* (Amaro, 2005), *C. gaudichaudii*, and *C. aeneus* (present study).

Cytogenetic data have provided important insights into interspecific comparisons in many groups, helping in evolutionary analyses. In cytogenetic studies of anurans, the NOR has been used as a valuable chromosome marker for the differentiation of species (Schmid et al., 2014) and even populations (Silva et al., 1999; Quinderé et al., 2009; Nascimento et al., 2019), although in several cases, the location of the NOR varies little among closely-related species (Busin et al., 2008; Cardozo et al., 2011). When we compared the karyotypes of *C. gaudichaudii* and *C. aeneus* based on diploid number, FN, NOR location, mapping of PcP190 satellite DNA and mapping of (CA)_15_ and (GATA)_8_ microsatellite clusters, no differences were found. Therefore, the cytogenetic traits described here to *C. gaudichaudii* and *C. aeneus* provide insufficient evidence for the differentiation of these two species.

The PcP190 satellite DNA was first described in the anuran *Physalaemus cuvieri* (Vittorazzi et al., 2011), and this satellite DNA family was subsequently detected in a number of anuran species, with a species-specific sequence variant being found in *C. gaudichaudii* (Vittorazzi *et al*., 2014). The data available on the PcP190 indicate that this satellite DNA family is a valuable chromosomal marker for karyotypic comparisons of the anurans. The chromosomal hybridization of PcP190 markers has revealed major interspecific differences in closely-related *Physalaemus* species, and differentiated the karyotypes of at least three *P. cuvieri* populations (Vittorazzi *et al*., 2014), later pointed as species (Lourenço et al., 2015). In the present analysis of *C. gaudichaudii* and *C. aeneus*, however, no clear differentiation of the karyotypes was found.

The chromosomes of *C. gaudichaudii* and *C. aeneus* present an accumulation of each analyzed microsatellite motifs, primarily in the subterminal regions of both arms, a pattern observed in the karyotype of a number of other anuran species (Peixoto et al., 2015, 2016; Ernetti et al., 2019). The enrichment of microsatellites in subterminal chromosomal regions may play a fundamental role in the stabilization and function of these regions in the eukaryotic chromosome (Buschiazzo and Gemmell, 2006; Richard et al., 2008; Torres et al., 2011), and has been found in several different vertebrate groups (Cioffi et al., 2011; Ruiz-Ruano et al., 2015; Peixoto *et al*., 2015, 2016; Oliveira et al., 2017).

The phylogenetic analyses based on the mitochondrial H1 fragment clustered all the specimens assigned to *C. gaudichaudii* and *C. aeneus* in a highly supported monophyletic group, which is the sister-clade of the *C. trachystomus+C. caramaschii+C. schmidti+C. werneri* clade. The *C. aeneus* specimens from Duque de Caxias, which is located in the same mountain range as the type locality of *C. aeneus*, clustered with a topotype of *C. aeneus* in the MP analysis, whereas they were grouped together with specimens of *C. gaudichaudii* in the ML analysis. Such uncertainty about the relationships of these specimens reinforces the taxonomic issues concerning *C. gaudichaudii* and *C. aeneus*. In addition, it agrees with the bPTP analysis, which assembled all the specimens assigned to *C. gaudichaudii* and *C. aeneus* in the same partition, as belonging to a single species. The genetic distance analysis was also congruent with these previous inferences, as low genetic divergence was found between specimens assigned to *C. gaudichaudii* and *C. aeneus*. While the genetic distances between *C. gaudichaudii* and *C. aeneus* ranged from 1.5% to 1.9% in the partial 16S rRNA gene, the other pairs of *Crossodactylus* species analyzed here varied between 7.4% and 10.7%, reflecting high levels of genetic diversification among valid species of this genus. These genetic distances estimated between *C. gaudichaudii* and *C. aeneus* were also below the threshold of 3% proposed by Fouquet et al. (2007) and Lyra et al. (2017) to flag candidate species based on this gene marker. Therefore, the genetic divergence among the specimens assigned to *C. gaudichaudii* and *C. aeneus* could represent population structure rather than interspecific variation.

In the past, other *nomen* had been synonymized with *C. gaudichaudii* (*Limnocharis fuscus* Bell, 1843; *Elosia vomerina* Girard, 1853; *Phyllobates brasiliensis* De Witte, 1830). Here, taken the cytogenetic and genetic results together with previous studies of internal and external morphology of tadpoles (Francioni and Carcerelli, 1993; Weber and Caramaschi, 2006; Silva-Soares et al., 2015) and biacoustic pattern (Pimenta et al., 2015), we notice that *C. gaudichaudii* and *C. aeneus* could not be differentiated, which supports the hypothesis that they correspond to the same taxonomic unit, with *C. aeneus* being a new junior synonym of *C. gaudichaudii*.

## Supplementary material

The following online material is available for this article:

Table S1 - Specimens of the *Crossodactylus* examined in collections to identify the specimens analyzed in the present work.

Table S2 - Specimens included in our analysis.

Figure S1 - Phylogenetic relationships inferred by Maximum Parsimony among the *Crossodactylus* species analyzed in the present study.

Figure S2 - Chromosomes of *C. gaudichaudii* and *C. aeneus* C-banded.
