# Supplementary material for: Cytogenetic and genetic data support Crossodactylus aeneus Müller, 1924 as a new junior synonym of C. gaudichaudii Duméril and Bibron, 1841 (Amphibia, Anura)
Source: Genet Mol Biol. 2021 Mar 22;44(2):e20200301. doi: 10.1590/1678-4685-GMB-2020-0301 (PMC7995990; doi:10.1590/1678-4685-GMB-2020-0301)
Supplement: Table S1 - [file 1415-4757-GMB-44-2-e20200301-s1.pdf]

Genetics and Molecular Biology
https://doi.org/10.1590/1678-4685-GMB-2020-0301

Supplementary Material to "Cytogenetic and genetic data support *Crossodactylus aeneus* Müller, 1924 as a new junior synonym of *C. gaudichaudii* Duméril and Bibron, 1841 (Amphibia, Anura)"

Table S1 - Specimens of the *Crossodactylus* examined in collections to identify the specimens analyzed in the present work.

Specimen	Locality	Voucher number
C. aeneus	Guapimirim, Rio de Janeiro, Brazil	MNRJ 30982; MNRJ 35120; MNRJ 35985–86; MNRJ 35944–49); ZSM 45, 47/1947, paratypes
C. gaudichaudii	Parque Lage, Rio de Janeiro, Brazil	MNRJ 35957–60

MNRJ (Museu Nacional do Rio de Janeiro); ZSM (Zoologische Staatssammlung München)
