# Supplementary material for: Cytogenetic and genetic data support Crossodactylus aeneus Müller, 1924 as a new junior synonym of C. gaudichaudii Duméril and Bibron, 1841 (Amphibia, Anura)
Source: Genet Mol Biol. 2021 Mar 22;44(2):e20200301. doi: 10.1590/1678-4685-GMB-2020-0301 (PMC7995990; doi:10.1590/1678-4685-GMB-2020-0301)
Supplement: Table S2 - [file 1415-4757-GMB-44-2-e20200301-s2.pdf]

Genetics and Molecular Biology
https://doi.org/10.1590/1678-4685-GMB-2020-0301

Supplementary Material to "Cytogenetic and genetic data support *Crossodactylus aeneus* Müller, 1924 as a new junior synonym of *C. gaudichaudii* Duméril and Bibron, 1841 (Amphibia, Anura)"

Table S2 - Specimens included in our analysis. Their localities and identification (ID) numbers, and GenBank numbers of their DNA sequences are indicated. **ZUEC:** Museum of Zoology "Professor Adão José Cardoso" of the University of Campinas, **SMRP:** Shirlei Maria Recco-Pimentel tissue collection, University of Campinas.

Species	Family	Locality	ID number	GenBank	Citation
Crossodactylus gaudichaudii	Hylodidae	Parque Lage, Rio de Janeiro, Rio de Janeiro, Brazil	ZUEC 17569<br>SMRP 186.4	MT893617	This work
Crossodactylus gaudichaudii	Hylodidae	Parque Lage, Rio de Janeiro, Rio de Janeiro, Brazil	ZUEC 17570<br>SMRP 186.5	MT893618	This work
Crossodactylus gaudichaudii	Hylodidae	Parque Lage, Rio de Janeiro, Rio de Janeiro, Brazil	ZUEC 17571<br>SMRP 186.6	MT893619	This work
Crossodactylus aeneus	Hylodidae	Parque Municipal do Taquara, Duque de Caxias, Rio de Janeiro, Brazil	ZUEC 17578<br>SMRP 374.1	MT893620	This work
Crossodactylus aeneus	Hylodidae	Parque Municipal do Taquara, Duque de Caxias, Rio de Janeiro, Brazil	ZUEC 17579<br>SMRP 374.2	MT893621	This work
Crossodactylus aeneus	Hylodidae	Parque Municipal do Taquara, Duque de Caxias, Rio de Janeiro, Brazil	ZUEC 17580<br>SMRP 374.3	MT893622	This work
Crossodactylus aeneus	Hylodidae	Barreira, Guapimirim, Rio de Janeiro, Brazil	ZUEC 20459<br>SMRP 374.12	MT893623	This work

**Species**	**Family**	**Locality**	**ID number**	**GenBank**	**Citation**
Crossodactylus aeneus	Hylodidae	Cachoeiras de Macacu, Rio de Janeiro, Brazil	vnbj99	Not deposited	Amaral et al. (2019)
Crossodactylus aeneus	Hylodidae	Cachoeiras de Macacu, Rio de Janeiro, Brazil	vjbj100	Not deposited	Amaral et al. (2019)
Crossodactylus aeneus	Hylodidae	Cachoeiras de Macacu, Rio de Janeiro, Brazil	-	KM390791	Chaves et al. (Unpublished)
Crossodactylus schmidti	Hylodidae	Aristobulo del Valle, Misiones, Argentina	-	HQ290948	Santos and Cannatella (2011)
Crossodactylus schmidti	Hylodidae	Aristobulo del Valle, Misiones, Argentina	-	AY843579	Faivovich et al. (2005)
Megaelosia goeldii	Hylodidae	Rio Beija Flor, Teresópolis, Rio de Janeiro, Brazil	-	DQ283072	Frost et al., (2006)
Hylodes phylodes	Hylodidae	Picinguaba, Ubatuba, São Paulo, Brazil	-	DQ283096	Frost et al., (2006)
Eupsophus roseus (outgroup)	Alsodidae	Huiliches, Neuquen, Argentina	-	JX204217	Blotto et al. (2012)
Eupsophus emiliopugini (outgroup)	Alsodidae	La Picada, Chile	-	JX204205	Blotto et al. (2012)
Eupsophus septentrionlis (outgroup)	Alsodidae	Los Queules, Chile	-	JX204218	Blotto et al. (2012)
Eupsophus vertebralis (outgroup)	Alsodidae	Queule, Chile	-	JX204220	Blotto et al. (2012)
Eupsophus calcaratus (outgroup)	Alsodidae	Chiloe, Chile	-	JX204200	Blotto et al. (2012)

Species	Family	Locality	ID number	GenBank	Citation
Eupsophus migueli (outgroup)	Alsodidae	Mehuin, Chile	-	JX204209	Blotto et al. (2012)
Eupsophus insularis (outgroup)	Alsodidae	Isla Mocha, Chile	-	JX204207	Blotto et al. (2012)
Eupsophus nahuelbutensis (outgroup)	Alsodidae	Piedra del Aguila, Chile	-	JX204212	Blotto et al. (2012)
Alsodes vanzolinii (outgroup)	Alsodidae	Ramadillas, Chile	-	JX204189	Blotto et al. (2012)
Alsodes verrucosus (outgroup)	Alsodidae	Puyehue, Chile	-	JX204192	Blotto et al. (2012)
Alsodes neuquensis (outgroup)	Alsodidae	Alumine, Neuquen, Argentina	-	JX204173	Blotto et al. (2012)
Alsodes nodosus (outgroup)	Alsodidae	Valparaiso, Petorca, Zapallar, Chile	-	JX204174	Blotto et al. (2012)
Alsodes valdiviensis (outgroup)	Alsodidae	Cordillera Pelada, Chile	-	JX204187	Blotto et al. (2012)
Alsodes gargola (outgroup)	Alsodidae	Futaleufu, Chile	-	JX204165	Blotto et al. (2012)
Alsodes pehuenche (outgroup)	Alsodidae	Mendoza, Valle Pehuenche, Argentina	-	JX204177	Blotto et al. (2012)
Alsodes barrioi (outgroup)	Alsodidae	Rucapehuen, Chile	-	JX204154	Blotto et al. (2012)
Alsodes tumutuosus (outgroup)	Alsodidae	Region Metropolitana, Santiago, La Parva, Chile	-	JX204185	Blotto et al. (2012)

References

Amaral C, Chaves A, Borges JV, Pereira F, Silva BM, Silva DA, Amorim A, Carvalho EF and Rocha C (2019) Amphibians on the hotspot: Molecular biology and conservation in the South American Atlantic Rainforest. PLoS One 14:e0224320.

Blotto BL, Nuñez JJ, Basso NG, Úbeda CA, Wheeler WC and Faivovich J (2013) Phylogenetic relationships of a Patagonian frog radiation, the *Alsodes* + *Eupsophus* clade (Anura: Alsodidae), with comments on the supposed para-phyly of *Eupsophus*. Cladistics 29:113-131.

Faivovich J, Haddad CFB, Garcia PCA, Frost DR, Campbell JA and Wheeler W (2005) Systematic review of the frog family Hylidae, with special reference Hylinae: Phylogenetic analysis and taxonomic revision. Bull Am Mus Nat Hist 294:1-240.

Frost DR, Grant T, Faivovich J, Bain RH, Haas A, Haddad CFB, Sá RO, Channing A, Wilkinson M, Donnellan SC *et al*. (2006) The Amphibian Tree of Life. Bull Am Mus Nat Hist 297:1-291.

Santos JC and Cannatella DC (2011) Phenotypic integration emerges from aposematism and scale in poison frogs. PNAS 108:6175-6180.
