# Supplementary material for: Cytogenetic and genetic data support Crossodactylus aeneus Müller, 1924 as a new junior synonym of C. gaudichaudii Duméril and Bibron, 1841 (Amphibia, Anura)
Source: Genet Mol Biol. 2021 Mar 22;44(2):e20200301. doi: 10.1590/1678-4685-GMB-2020-0301 (PMC7995990; doi:10.1590/1678-4685-GMB-2020-0301)
Supplement: Figure S1 - [file 1415-4757-GMB-44-2-e20200301-s3.pdf]

Genetics and Molecular Biology
https://doi.org/10.1590/1678-4685-GMB-2020-0301

Supplementary Material to "Cytogenetic and genetic data support *Crossodactylus aeneus* Müller, 1924 as a new junior synonym of *C. gaudichaudii* Duméril and Bibron, 1841 (Amphibia, Anura)"

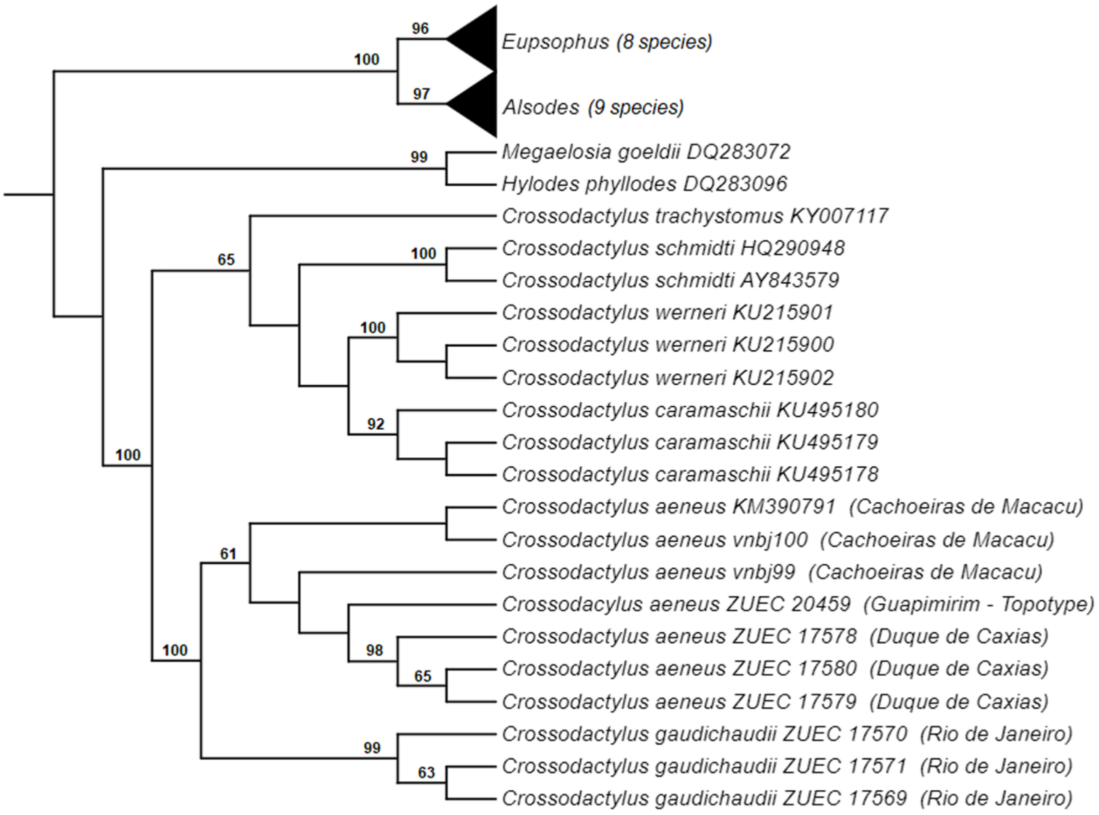

Figure S1 - Phylogenetic relationships among the *Crossodactylus* species analyzed in the present study, inferred by maximum parsimony analysis of a 2,369-bp H1 mitochondrial DNA sequence matrix. The numbers at each node indicate the bootstrap values (values below 50 have been omitted).
