# Supplementary material for: Cytogenetic and genetic data support Crossodactylus aeneus Müller, 1924 as a new junior synonym of C. gaudichaudii Duméril and Bibron, 1841 (Amphibia, Anura)
Source: Genet Mol Biol. 2021 Mar 22;44(2):e20200301. doi: 10.1590/1678-4685-GMB-2020-0301 (PMC7995990; doi:10.1590/1678-4685-GMB-2020-0301)
Supplement: Figure S2 - [file 1415-4757-GMB-44-2-e20200301-s4.pdf]

Genetics and Molecular Biology
https://doi.org/10.1590/1678-4685-GMB-2020-0301

Supplementary Material to "Cytogenetic and genetic data support *Crossodactylus aeneus* Müller, 1924 as a new junior synonym of *C. gaudichaudii* Duméril and Bibron, 1841 (Amphibia, Anura)"

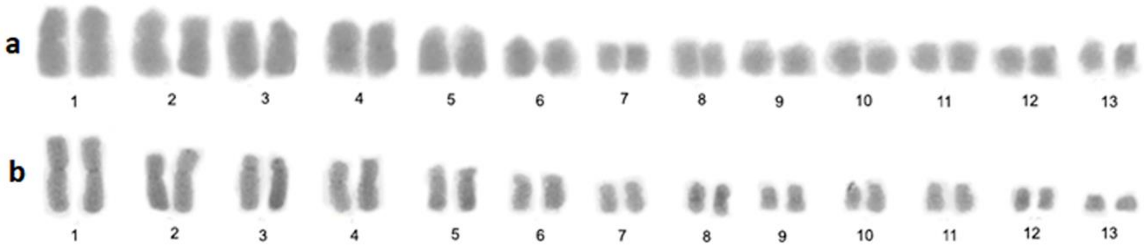

Figure S2 - Chromosomes of *C. gaudichaudii* (a) and *C. aeneus* (b) C-banded.
